# Estimates of the Prevalence of Pandemic (H1N1) 2009, United States, April–July 2009

**DOI:** 10.3201/eid1512.091413

**Published:** 2009-12

**Authors:** Carrie Reed, Frederick J. Angulo, David L. Swerdlow, Marc Lipsitch, Martin I. Meltzer, Daniel Jernigan, Lyn Finelli

**Affiliations:** Centers for Disease Control and Prevention, Atlanta, Georgia, USA (C. Reed, F.J. Angulo, D.L. Swerdlow, M.I. Meltzer, D. Jernigan, L. Finelli); Harvard School of Public Health, Boston, Massachusetts, USA (M. Lipsitch)

**Keywords:** Influenza, pandemic, pandemic (H1N1) 2009, viruses, dispatch, expedited

## Abstract

Through July 2009, a total of 43,677 laboratory-confirmed cases of influenza A pandemic (H1N1) 2009 were reported in the United States, which is likely a substantial underestimate of the true number. Correcting for under-ascertainment using a multiplier model, we estimate that 1.8 million–5.7 million cases occurred, including 9,000–21,000 hospitalizations.

Human cases of influenza A pandemic (H1N1) 2009 were first identified in the United States in April 2009 (*1*,*2*). By the end of July, >40,000 laboratory-confirmed infections had been reported, representing only a fraction of total cases. Persons with influenza may not be included in reported counts for a variety of reasons, including the following: not all ill persons seek medical care and have a specimen collected, not all specimens are sent to a public health laboratory for confirmatory testing with reverse transcription–PCR (RT-PCR; rapid point-of-care testing cannot differentiate pandemic [H1N1] 2009 from other strains), and not all specimens will give positive results because of the timing of collection or the quality of the specimen. To better estimate the prevalence of pandemic (H1N1) 2009 during April–July 2009 in the United States, we created a simple multiplier model that adjusts for these sources of under-ascertainment.

## The Study

Through July 23, 2009, a total of 43,677 laboratory-confirmed infections with pandemic (H1N1) 2009 had been reported in the United States by the 50 states and the District of Columbia, including 5,009 hospitalizations and 302 deaths. To estimate the total number of cases of pandemic (H1N1) 2009, we built a probabilistic multiplier model that adjusts the count of laboratory-confirmed cases for each of the following steps: medical care seeking (A), specimen collection (B), submission of specimens for confirmation (C), laboratory detection of pandemic (H1N1) 2009 (D), and reporting of confirmed cases (E) (Figure). This approach has been used to calculate the underrecognized impact of foodborne illness in the United States (*3*).

**Figure:**
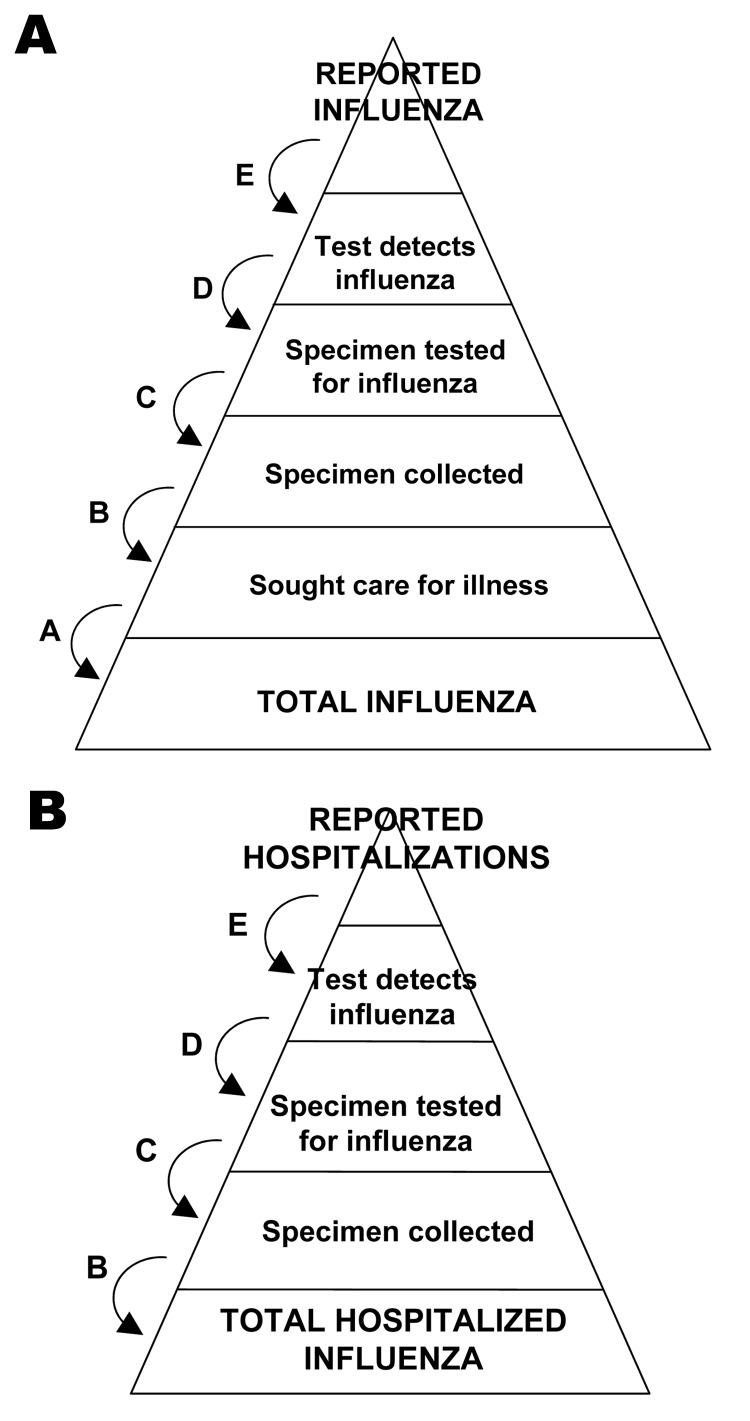
Schematic of the steps involved in adjusting counts of reported cases of pandemic (H1N1) 2009 to estimate total cases.

At each step, we identified a range of proportions observed in prior published studies and recent surveys and investigations of pandemic (H1N1) 2009. These include 2 unpublished community surveys on influenza-like illness (ILI) and health-seeking behavior, the 2007 Behavioral Risk Factor Surveillance Survey conducted in 10 states and repeated in the same states during May 2009, and field investigations conducted during early outbreaks of pandemic (H1N1) 2009 in Chicago and Delaware (Technical Appendix; [*4*]). We theorized that, given recommendations for testing, patients hospitalized with pandemic (H1N1) 2009 would more likely have been tested and their cases reported than would outpatients. We therefore stratified our model between hospitalized and nonhospitalized cases (Figure). For hospitalized patients, we used larger estimates of the proportion of specimens collected, tested, and reported, which resulted in smaller multiplier values (Table 1). We also adjusted for the fact that early in the epidemic physicians and health departments were encouraged to collect clinical specimens from all suspect case-patients with ILI and forward them for confirmatory testing with RT-PCR. By May 12, due to the increasing number of cases and the demands on public health laboratories, guidance for confirmatory testing was revised to focus on hospitalized patients. We therefore used a lower estimate for the proportion of specimens collected from patients with mild illness after that date, effectively increasing the multiplier for those patients (Table 1).

**Table 1:** Model parameters and sources of data included in the model estimating prevalence of pandemic (H1N1) 2009, United States, April–July 2009*

Parameter		Observed value	Source	Ranges included in the model, %	
				Not hospitalized	Hospitalized
A	Proportion of persons with influenza who seek medical care, %	42	2007 BRFSS, 9 states†	42–58	100
		52–55	2009 ILI survey, 10 states†		
		49–58	Delaware university survey		
		52	Chicago community survey		
B	Proportion of persons seeking care with a specimen collected, %	25	2007 BRFSS, 9 states†	19–34	40–75
		22–28	2009 ILI survey, 10 states†		
		19–34	Delaware university survey		
C	Proportion of specimens collected that are sent for confirmatory testing, %	26 (through May 3)	Delaware university survey	20–30 (through May 12); 5–15 (after May 12)	50–90
D	Test detects influenza		Published studies	90–100	90–100
E	Proportion of confirmed cases reported to CDC		Assumption	95–100	95–100
	No. reported cases	43,677	Reports to CDC through July 23, 2009	4,759 (through May 12); 33,909 (after May 12)	5,009

*BFRSS, Behavioral Risk Factor Surveillance Survey; ILI, Influenza-like illness; CDC, Centers for Disease Control and Prevention. States include California, Colorado, Connecticut, Georgia, Maryland, Minnesota, New Mexico, New York, Oregon, and Tennessee. †Parameter estimates and sources are described in further detail in the Technical Appendix.

Multipliers were calculated as the simple inverses of the proportions at each step. We accounted for variability and uncertainty in model parameters by using a probabilistic (Monte Carlo) approach (built by using SAS version 9.2; SAS Institute, Cary, NC, USA). For each parameter included in the model, we used uniform probability distributions that covered a range of minimum to maximum values, from which the model randomly sampled 10,000 iterations (Technical Appendix). We generated median, upper, and lower 90% values for the number of total illnesses and hospitalizations.

To further divide estimated cases into age groups, we applied the age distribution of confirmed cases and hospitalizations as reported to the US Centers for Disease Control and Prevention through July 23, 2009 (Technical Appendix), and calculated overall and age-specific incidence of illness and hospitalization, based on the US Census monthly population estimates for May 2009. We did not have age-specific parameter estimates, and thus did not stratify by age group within the model. This approach may not fully capture differences in the probability of ascertainment by age.

Using this approach, between April and July 2009, we estimate that the median multiplier of reported to estimated cases was 79; that is, every reported case of pandemic (H1N1) 2009 may represent 79 total cases, with a 90% probability range of 47–148, for a median estimate of 3.0 million (range 1.8–5.7 million) symptomatic cases of pandemic (H1N1) 2009 in the United States. Likewise, we estimate that every hospitalized case of pandemic (H1N1) 2009 that was reported may represent a median of 2.7 total hospitalized persons (90% range 1.9–4.3). This represents a median estimate of 14,000 (range 9,000–21,000) hospitalizations (Table 2) and thus an estimated ratio of hospitalizations to total symptomatic cases of 0.45% (range 0.16%–1.2%).

**Table 2:** Estimates of pandemic (H1N1) 2009–related cases and rates of illness and hospitalization by age distribution of confirmed case-patients, United States, April–July 2009

Parameter	Estimated no. case-patients			Estimated rate/100,000*	
	Median	90% range		Median	90% range
Total no. case-patients by age group, y†	3,052,768	1,831,115–5,720,928		997	598–1,868
0–4	397,033	238,149–744,045		1,870	1,122–3,505
5–24	1,820,284	1,091,845–3,411,237		2,196	1,317–4,115
25–49	612,862	367,608–1,148,511		577	346–1,081
50–64	180,297	108,146–337,879		319	192–599
>65	42,292	25,368–79,256		107	64–201
No. hospitalized case-patients by age group, y	13,764	9,278–21,305		4.5	3.0–7.0
0–4	2,768	1,866–4,285		13.0	8.8–20.2
5–24	4,991	3,364–7,725		6.0	4.1–9.3
25–49	3,440	2,319–5,324		3.2	2.2–5.0
50–64	1,912	1,289–2,959		3.4	2.3–5.2
>65	654	441–1,012		1.7	1.1–2.6
Multiplier					
Hospitalized	2.7	1.7–4.5		–	–
Nonhospitalized	79	47–148		–	–
Through May 12	33	23–49		–	–
After May 12	84	50–163		–	–

*United States Population Estimates, 2009. †Age distributions from line list and aggregate reports of laboratory-confirmed cases and hospitalizations to the Centers for Disease Control and Prevention through July 23, 2009.

We also estimate that incidence of pandemic (H1N1) 2009 over the first 4 months of the pandemic in the United States ranged from a median of 107/100,000 in persons >65 years of age, to 2,196/100,000 in persons 5–24 years of age (Table 2). The incidence of hospitalization was estimated to be highest in young children <5 years of age (median 13.0/100,000, 90% range 8.8–20.2).

## Conclusions

We demonstrate that the reported cases of laboratory confirmed pandemic (H1N1) 2009 are likely a substantial underestimation of the total number of actual illnesses that occurred in the community during the spring of 2009. We estimate that through July 23, 2009, from 1.8 million to 5.7 million symptomatic cases of pandemic (H1N1) 2009 occurred in the United States, resulting in 9,000–21,000 hospitalizations. We did not estimate the number of deaths directly from our model, but among reports of laboratory-confirmed cases though July 23, the ratio of deaths to hospitalizations was 6%. When applying this fraction to the number of hospitalizations calculated from the model—that is, by assuming that deaths and hospitalizations are underreported to the same extent—we obtain a median estimate of 800 deaths (90% range 550–1,300) during this same period. Because this assumption has several limitations (*5*), more sophisticated models are also being developed to better understand the severity of the US epidemic in the spring of 2009, including intensive care unit admissions and deaths (*6*).

Our analysis involves several assumptions. Data for parameter estimates were collected in limited periods and areas and thus may not be fully representative of the entire United States. To account for some of this uncertainty, a range of values was included for each proportion. Additional data from surveys of health-seeking behavior, physician testing practices, and policies for confirmatory testing at public health laboratories could help refine the parameter estimates. In addition, parameters were obtained from studies of persons with ILI, defined as fever with cough or sore throat. Persons with milder illness may be less likely to seek care or be tested, and thus may not be fully captured in these estimates. Likewise, in some heavily affected areas, the size of the outbreak quickly exceeded the capacity to ascertain and test case-patients. Thus, our results may reflect a conservative estimate of total cases.

As pandemic (H1N1) 2009 continues to spread through the United States and the world, laboratory-confirmed cases will continue to greatly underestimate the number of actual cases that occur. Surveillance for influenza does not traditionally rely on complete case ascertainment, which would be impractical, but on focused case ascertainment with well-characterized surveillance systems and special studies. Unfortunately, relying on laboratory-confirmed cases limits the ability to understand the full impact and severity of the epidemic, especially when severe cases are more likely to be recognized (*5*).

This model provides a relatively quick and simple approach to estimate the human health impact of the epidemic in advance of more rigorous analysis of surveillance and health care data that will be available over the next few years. Health systems and infrastructure may be unprepared in the short-term if plans are based on a number of confirmed cases that substantially underestimates the impact of the epidemic. We estimate that the total number of pandemic (H1N1) 2009 cases in the United States during April–July 2009 may have been up to 140× greater than the reported number of laboratory confirmed cases. A spreadsheet version of the model has been developed and is available online (www.cdc.gov/h1n1flu/tools). Using this tool, health officials and policy makers could adjust the model parameters to represent their local experience, which may provide useful estimates of the prevalence of pandemic (H1N1) 2009 in their areas and help plan for a subsequent wave of the epidemic in the fall and winter months of 2009–2010.

## Supplementary Material

Technical Appendix Sources for Parameter Estimates
