# Supplementary material for: Estimates of the Prevalence of Pandemic (H1N1) 2009, United States, April–July 2009
Source: Emerg Infect Dis. 2009 Dec;15(12):2004–7. doi: 10.3201/eid1512.091413 (PMC3375879; doi:10.3201/eid1512.091413)
Supplement: Technical Appendix — Sources for Parameter Estimates [file 09-1413_Techapp-s1.pdf]

Estimates of the Prevalence of Pandemic (H1N1) 2009, United States, April–July 2009

Technical Appendix

Sources for Parameter Estimates

Parameters were estimated from a number of different surveillance systems and special investigations. These sources are briefly described below.

2007 Behavioral Risk Factor Surveillance Survey (BRFSS)

The BRFSS, a random-digit-dialed telephone survey was established by the Centers for Disease Control and Prevention (CDC) and state health departments in 1984 to obtain a representative sample of adults ≥18 years of age in each state and the District of Columbia. The BRFSS collects information annually on a core set of health behaviors, and also may include a variety of additional public health modules. In 2007, a module on influenza-like illness (ILI) was included in 9 states, designed to assess the incidence of ILI, health-seeking behavior, physician diagnosis of influenza, and treatment of influenza with antiviral medications. The 9 participating states included California, Colorado, Connecticut, Georgia, Maryland, Minnesota, New Mexico, Oregon and Tennessee. A total of 51,249 persons ≥18 years of age participated in these states during the 12 month survey. Analysis of BRFSS data involves weighting for the probability of selection of a telephone number, the number of adults in a household, and the number of telephones in a household, as well as to reflect the age and gender distribution of the underlying population.

2009 ILI Community Survey

In May 2009, after the identification of pandemic (H1N1) 2009 in the United States, a random-digit dialed telephone survey sampled similarly to the BRFSS was conducted using only the ILI module from the 2007 BRFSS and some limited demographic information. Respondents were adults ≥18 years of age living in the same 9 states where the ILI module was included

during the 2007 BRFSS plus New York State. Participants were asked the same set of questions included in the ILI module during the 2007 BRFSS, including ILI in the past month, care-seeking behavior, receipt of antiviral treatment, and influenza vaccination. Participants were also asked the same questions about all members of their household. A total of 1,788 adults responded during a 3-week period. As with analysis of the BRFSS, data were weighted for the probability of selection and the age and gender distribution of the population.

Chicago Community Survey

Illness with pandemic (H1N1) 2009 virus was first documented in Chicago in late April 2009. By early May, a community in northeast Chicago accounted for approximately one third of the city's confirmed cases. To further investigate this early outbreak of pandemic (H1N1) 2009 in Chicago and better characterize community transmission, a community survey was conducted in this neighborhood. Using a multistage cluster design, investigators obtained a representative sample of households in six census areas and then administered a standardized questionnaire to enrolled households. A total of 240 households and 643 persons were enrolled, including adults and children.

Delaware University Survey

A large outbreak of pandemic (H1N1) 2009 occurred on a university campus in Delaware in late April 2009. Data were available on clinic visits and influenza testing among students from the campus health center. To further describe the extent of the outbreak and effect of illness on campus, an online survey was conducted to assess health-seeking behaviors, influenza vaccination status, risk factors for illness, prevention practices, and measures to reduce transmission. A total of 6,049 students (32% response rate) and 1,401 faculty/staff (24% response rate) responded to the online survey over a 1-week period.

Values for Parameter Estimates

A) We estimate that between 42% and 58% of persons with influenza-like illness sought medical care for their illness. The low of 42% was calculated through a weighted analysis of the 2007 BRFSS, during an annual influenza season before the emergence of pandemic (H1N1) 2009. A similar survey conducted in the same states in May 2009 during early pandemic (H1N1) 2009 outbreaks found that a slightly higher proportion (52%) of respondents with ILI reported

seeking medical care for their illness. Participants in this survey were also asked about other members of their household, and 55% of household members with ILI in May 2009 were reported to have sought medical care. Similarly, a university-wide survey in Delaware indicated that 58% of students with ILI and 49% of faculty and staff with ILI reported that they sought medical care for their illness. Finally, 52% of persons with ILI included in a community household survey in a Chicago neighborhood reported seeking medical attention.

These values are all calculated from self-reported medical visits following ILI, and thus may overestimate care-seeking behavior. Some recent studies on the accuracy of self-reported medical visits find that self-reported care seeking in population surveys tends to overstate the actual number of medical visits seen in the same community (*1*,*2*). If the true value for this parameter is lower, it would result in a higher multiplier and thus a higher estimate of cases than we present.

B) Of patients who reported seeking outpatient care, we estimate that between 22% and 34% had a swab taken by the physician for an influenza test. Weighted analysis of the BRFSS data from 2007 indicated that 25% of respondents reported having been swabbed for an influenza test. A similar analysis of the data from 2009 found that 22% of respondents and 28% of their household members reportedly were swabbed for an influenza test. During the Delaware investigation, 34% of students and 30% of faculty who sought medical care reported that they were swabbed for an influenza test. All of these values come from self-reports of influenza testing. Data from the campus health center were also available from the Delaware investigation, and these showed that 19% of patients presenting with febrile respiratory illness had a specimen collected for a rapid influenza test. Anecdotally, physicians continued collecting clinical specimens from patients with ILI for rapid antigen tests, even though not all were sent for further subtyping and confirmation with reverse transcription–PCR (RT-PCR).

During 2 recent influenza seasons, capture-recapture analyses were performed in areas that had two independent surveillance systems operating concurrently for influenza-associated hospitalizations in children <5 years of age. These analyses found that laboratory-based surveillance from routine clinical testing identified 38%–39% of all pediatric influenza-related hospitalizations, and prospective surveillance and testing of all children with acute febrile illness still only identified 69%–74% of children with influenza (*3*,*4*). Although no similar data are

available on surveillance for influenza-related hospitalizations in older children and adults, we would hypothesize that detection may even be lower for older age groups. Given the variety of approaches to surveillance between states and hospitals, we include a fairly broad range of 40%–75%.

C) In the United States, health departments were encouraged to seek laboratory confirmation of suspect cases with RT-PCR at federal or state public health laboratories early in the epidemic; however, as the epidemic progressed and transmission became widespread, complete case ascertainment became prohibitive, and by May 12, 2009, physicians were recommended to primarily test patients in special risk groups or those with severe illness. Data from the campus health clinic during the Delaware outbreak (before May 12) indicate that ≈26% of specimens collected by physicians may have been sent to the state for confirmatory testing; we included a range of 20%–30% in the model for this period. The proportion of samples sent for confirmatory testing likely decreased after activity became more widespread and testing recommendations were changed to focus on severe cases. Through May 12, hospitalized cases made up 4% of all the cases reported, but accounted for 12% of the reported cases after May 12. Thus, confirmatory testing of non-severe cases may have decreased three-fold following the change in recommendations. We included a 2×–4× lower range in our model for cases reported after May 12.

Likewise in hospitalized patients, although many physicians continued to collect clinical specimens for rapid point-of-care testing, not all may have been forwarded to public health laboratories for confirmation of pandemic (H1N1) 2009. We do not have any direct estimates of the proportion of specimens sent for confirmation during this period. We do know that in some locations with large outbreaks, specimens from hospitalized patients were only sent for confirmation if the patient had a positive rapid influenza test result. Given the sensitivity of rapid diagnostic tests for pandemic (H1N1) 2009 in early studies (*5*,*6*), this finding may represent as few as 50% of true influenza cases. Due to the uncertainty in this parameter estimate, we include a broad range of 50%–90%.

D) Although RT-PCR is known to have a high sensitivity, the ability of the test to detect influenza in a clinical specimen may be influenced by the quality of the specimen, specimen handling, timing of collection, or age of the patient (*7*,*8*). We included a range of 90%–100%

detection, which includes a range of sensitivities for RT-PCR seen in published studies. One previous study based on Canadian influenza surveillance, however, estimated that the sensitivity of testing given the quality of specimens collected and submitted in practice may be lower (*9*). If the true proportion is lower, this would lead to a higher multiplier and thus a higher estimate of total cases than we present.

E) Finally, due to miscommunication or delays in reporting, some patients with a positive test result may not be reported by the state health department to CDC and thus included in official case counts. Although we do not have any direct estimates of this fraction, we included a range of 95%–100% reporting.

Data on ascertainment of fatal cases are even more limited. Consequently, we chose not to estimate fatal cases directly in the model, but include a rough extrapolation based on the ratio of reported deaths to reported hospitalizations. More sophisticated models are being developed to estimate the severity of pandemic (H1N1) 2009 during the spring, including admission to an intensive care unit and death (*10*)

References

1. Baker MG, Wilson N, Huang QS, Paine S, Lopez L, Bandaranayake D, et al. Euro Surveill. 2009 Aug 27;14(34). pii: 19319. PubMed

2. Metzger KB, Hajat A, Crawford M, Mostashari F. How many illnesses does one emergency department visit represent? Using a population-based telephone survey to estimate the syndromic multiplier. MMWR Morb Mortal Wkly Rep. 2004;53(Suppl):106–11. PubMed

3. Grijalva CG, Craig AS, Dupont WD, Bridges CB, Schrag ST, Iwane MK, et al. Estimating influenza hospitalizations among children. Emerg Infect Dis. 2006;12:103–9. PubMed

4. Grijalva CG, Weinberg GA, Bennett NM, Staat MA, Craig AS, Dupont WD, et al. Estimating the undetected burden of influenza hospitalizations in children. Epidemiol Infect. 2007;135:951–8. PubMed DOI: 10.1017/S095026880600762X

5. Centers for Disease Control and Prevention. Evaluation of rapid influenza diagnostic tests for detection of novel influenza A (H1N1) virus—United States, 2009. MMWR Morb Mortal Wkly Rep. 2009;58:826–9. PubMed

6. Faix DJ, Sherman SS, Waterman SH. Rapid-test sensitivity for novel swine-origin influenza A (H1N1) virus in humans. N Engl J Med. 2009;361:728–9. PubMed DOI: 10.1056/NEJMc0904264

7. Carrat F, Vergu E, Ferguson NM, LeMaitre M, Cauchemez S, Leach S, et al. Time lines of infection and disease in human influenza: a review of volunteer challenge studies. Am J Epidemiol. 2008;167:775–85. PubMed DOI: 10.1093/aje/kwm375

8. Wallace LA, Collins TC, Douglas JD, McIntyre S, Millar J, Carman WF. Virological surveillance of influenza-like illness in the community using PCR and serology. J Clin Virol. 2004;31:40–5. PubMed DOI: 10.1016/j.jcv.2003.12.003

9. Schanzer DL, Garner MJ, Hatchette TF, Langley JM, Aziz S, Tam TW. Estimating sensitivity of laboratory testing for influenza in Canada through modelling. PLoS One. 2009;4:e6681. PubMed DOI: 10.1371/journal.pone.0006681

10. Presanis AM, Lipsitch M, De Angelis D, New York City Swine Flu Investigation Team, Hagy A, Reed C, et al. The severity of pandemic H1N1 influenza in the United States, April–June 2009. PLoS Currents Influenza. 2009 [cited 2009 Oct 23]. Available from http://knol.google.com/k/anne-m-presanis/the-severity-of-pandemic-h1n1-influenza/agr0htar1u6r/16#

Table. Age distribution of reported cases, hospitalizations, and deaths, United States, April– July 2009

Age group, y	Cases,* %	Hospitalizations,* %	Deaths,* %
0–4	13.0	20.1	2.5
5–24	59.6	36.3	17.4
25–49	20.1	25.0	44.9
50–64	5.9	13.9	25.7
≥65	1.4	4.7	9.4

*Age recorded for 85% of cases, 95% of hospitalizations, and 91% of deaths reported through July 23, 2009.

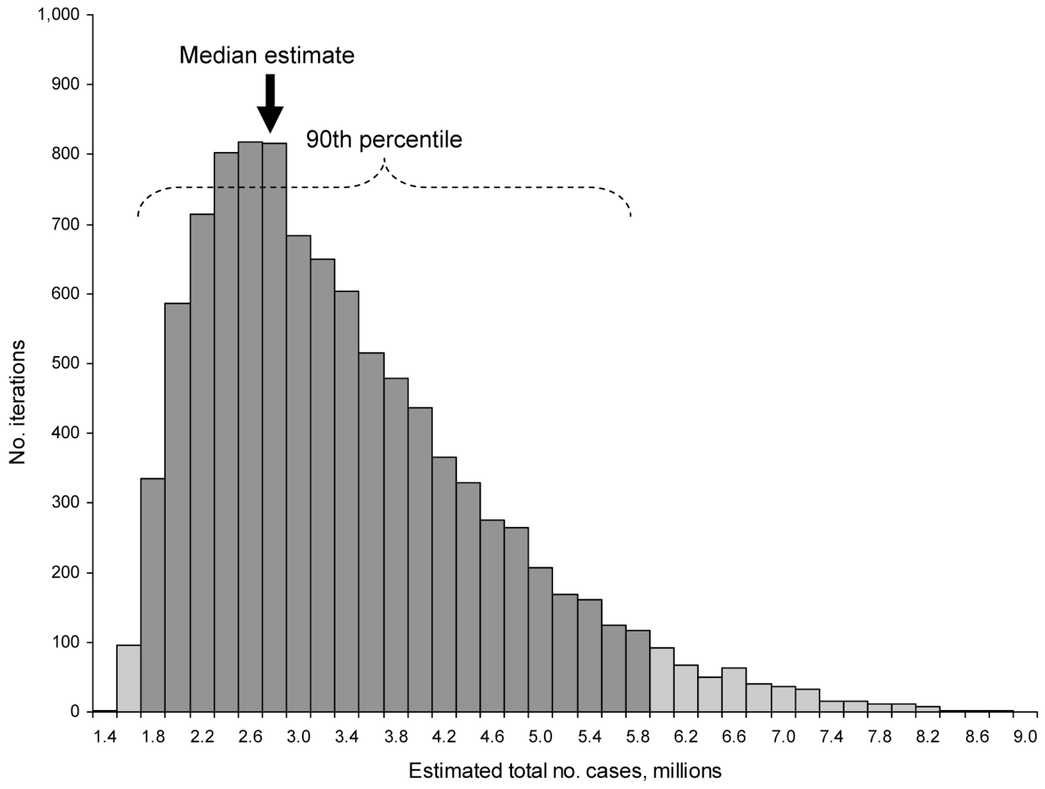

Figure. Distribution of the estimated number of total cases of pandemic (H1N1) 2009 (in millions) calculated from Monte Carlo simulation (10,000 iterations showing median estimate and 90% range).
